# How effective is ketogenic diet in sleep disorders ?

**DOI:** 10.1192/j.eurpsy.2024.1617

**Published:** 2024-08-27

**Authors:** N. Kouki, A. maamri, N. kouki, M. A. zaafrane, A. Hajri, H. zalila

**Affiliations:** Outpatient psychiatry department, Razi hospital, manouba, Tunisia

## Abstract

**Introduction:**

Sleep disorders vary widely and its treatment are based on a combination of life style changes and pharmacological therapy adapted to the primer health issue. Ketogenic diet has shown not only its efficacy in different health conditions, but it is also becoming a popular health trend. Could the therapeutic spectrum of ketogenic diet cover sleep disturbances ?

**Objectives:**

The aim of our study is to evaluate the effect of ketogenic diet on sleep disorders

**Methods:**

To identify relevant studies ,our literature review was based on the Pubmed interface and adapted for 2 databases : science direct and google scholar. We used the following key words ( ketogenic diet [meSH terms]) and (sleep disorders [meSH terms]).

**Results:**

Our research revealed 14 articles published between 2012 and 2022. We selected 8 which corresponded to the purpose of our review. The ketogenic diet affects sleep hemostasis indirectly. In fact, this diet is associated with weight loss and therefore reduction of metabolic and cardiovascular complications disturbing sleep quality.  From a neurobiological perspective, this regimen based on limited carbohydrates is associated with a low Tryptophan intake which is the precursor of melatonin. But on the other hand, Ketone bodies trigger adenosine activity which promotes melatonin liberation, the sleep inducing hormone.

**Conclusions:**

Ketogenic diet modulates melatonin activity therefore affects sleep architecture. Meanwhile, Its impact on sleep disorders is still controversed due to the variation of its pathophysiological mechanisms.

**Disclosure of Interest:**

None Declared

